# New approaches in gene therapy for sickle cell disease, moving in vivo

**DOI:** 10.1002/hem3.43

**Published:** 2024-03-12

**Authors:** David G. Kent

**Affiliations:** ^1^ Department of Biology, York Biomedical Research Institute University of York York UK

The last few years have witnessed an absolute explosion of new approaches for gene therapy and the number of ongoing clinical trials has been on a similarly upward trajectory. Alongside this wave of activity, there have been many developments in novel methods to undertake the gene editing with lipid nanoparticles, extracellular vesicles, CRISPR‐Cas, and nucleic acids all emerging as potentially useful modalities. These latter approaches all hold the possibility of being combined with traditional in vitro methods of altering the genome (as currently done in the vast majority of clinical trials), but also open up a huge range of opportunities for exploring new delivery methods including the possibility of moving to direct in vivo treatment. This is a critical area for new developments to take place because, if achievable, would be a major advance on the path to democratizing access to gene therapies across the world.

Many current gene therapies for cells of the blood system involve the harvesting of a patient's blood stem cells, the in vitro gene editing of those cells (typically by viral delivery of genetic material) and the transplantation of the gene‐edited drug product back into the patient whereby the edited donor stem cells repopulate the host. In general, this process has enjoyed enormous success with curing the underlying genetic disorder, although it has also been plagued by its share of trouble along the way, including the subsequent development of hematological malignancies in some patients. As a result, the production and quality control of the aforementioned drug product is substantial and results in equally substantial cost implications which inevitably limit the future application of successful gene therapies.^1^ Therapies that might remove the in vitro component, which involves large granulocyte macrophage progenitor‐level virus production and extensive quality control regimens, would therefore be of great utility and the field of in vivo therapies has understandably garnered intense interest.

Recently, a paper in *Blood* emerged from Andre Lieber's group which gives a glimpse into what in vivo gene therapy might look like.^2^ It shows that in vivo treatment with a prime editor can correct the sickle cell mutation and ameliorate disease symptoms without the need for an in vitro transduction step and without undesired off target effects. The approach does not require ex vivo manipulation, transplantation or myeloablation which makes it very attractive in concept.

The approach used by the Lieber group is tantalizingly simple—there is a single injection of a nonintegrating prime‐editor‐expressing viral vector accompanied by low dose drug selection in vivo. The result was correction of ∼40% of the alleles in hematopoietic stem cells (HSCs) with accompanying resolution of the sickle cell disease phenotype in the CD46/Townes mouse model of disease. The off‐target effects are expected to be reduced (although not extensively tested on a per cell basis in the paper) because the prime editing has three independent DNA‐binding events which should minimize the chances that it introduces changes in different genomic regions. Also, in comparison to general CRISPR/Cas9 approaches, prime editing does not introduce double stranded breaks. While it is still at the mouse model stage, this approach achieves the important benchmark of successful in vivo editing of functional (i.e., secondarily transplantable) HSCs—something that is essential for long term success of a gene‐edited graft of cells in patient populations.

While these results from the Lieber lab are incredibly exciting and overlay onto similarly exciting activity in the clinical trials space, it is important to remember that these studies are at the proof‐of‐concept stage in mouse models and will require extensive work before this can become a clinical reality. The authors are very up front about these challenges in their discussion, highlighting both the potential for immune system reaction to the injected substances and the need to optimize or eliminate the drug selection required to give the edited cells a selective advantage.

If achievable, a safe in vivo gene editing protocol would have the enormous attractiveness of being much less expensive to produce and require much less specialized posttransplantation processing and monitoring. These aspects would make such therapies an absolute game‐changer for countries where a single curative visit to a treatment facility would be the only option for large scale and affordable adoption. In genetics disorders such as sickle cell disease where the largest burden of disease matches to resource‐poor regions of the world, this becomes even more important to explore.

## AUTHOR CONTRIBUTIONS

David G. Kent is the sole author of this article.

## CONFLICT OF INTEREST STATEMENT

The author declares no relevant conflict of interest.

## FUNDING

The DGK lab is supported by a CRUK Programme Foundation Award and the UK Medical Research Council.

## Data Availability

Data sharing is not applicable to this article as no new data were created or analyzed in this study.
