# Correction to “New approaches in gene therapy for sickle cell disease, moving in vivo”

**DOI:** 10.1002/hem3.107

**Published:** 2024-06-26

Kent DG. New approaches in gene therapy for sickle cell disease, moving in vivo. *HemaSphere*. 2024;8:e43.

In paragraph 2, the final sentence included the text “…involves large granulocyte‐macrophage progenitor‐level virus protection…”, which was incorrect. This should have read “Therapies that might remove the in vitro component, which involves substantial good manufacturing practice virus production and extensive quality control regimens, would therefore be of great utility and the field of in vivo therapies has understandably garnered intense interest”.

We apologize for this error.

